# Nurse-Physician Communication Team Training in Virtual Reality Versus Live Simulations: Randomized Controlled Trial on Team Communication and Teamwork Attitudes

**DOI:** 10.2196/17279

**Published:** 2020-04-08

**Authors:** Sok Ying Liaw, Sim Win Ooi, Khairul Dzakirin Bin Rusli, Tang Ching Lau, Wilson Wai San Tam, Wei Ling Chua

**Affiliations:** 1 Alice Lee Centre for Nursing Studies National University of Singapore Singapore Singapore; 2 National University Hospital Singapore Singapore; 3 Yong Loo Lin School of Medicine National University of Singapore Singapore Singapore

**Keywords:** interprofessional education, team training, nurse-physician communication, virtual reality, simulation

## Abstract

**Background:**

Interprofessional team training is needed to improve nurse-physician communication skills that are lacking in clinical practice. Using simulations has proven to be an effective learning approach for team training. Yet, it has logistical constraints that call for the exploration of virtual environments in delivering team training.

**Objective:**

This study aimed to evaluate a team training program using virtual reality vs conventional live simulations on medical and nursing students’ communication skill performances and teamwork attitudes.

**Methods:**

In June 2018, the authors implemented nurse-physician communication team training using communication tools. A randomized controlled trial study was conducted with 120 undergraduate medical and nursing students who were randomly assigned to undertake team training using virtual reality or live simulations. The participants from both groups were tested on their communication performances through team-based simulation assessments. Their teamwork attitudes were evaluated using interprofessional attitude surveys that were administered before, immediately after, and 2 months after the study interventions.

**Results:**

The team-based simulation assessment revealed no significant differences in the communication performance posttest scores (*P*=.29) between the virtual and simulation groups. Both groups reported significant increases in the interprofessional attitudes posttest scores from the baseline scores, with no significant differences found between the groups over the 3 time points.

**Conclusions:**

Our study outcomes did not show an inferiority of team training using virtual reality when compared with live simulations, which supports the potential use of virtual reality to substitute conventional simulations for communication team training. Future studies can leverage the use of artificial intelligence technology in virtual reality to replace costly human-controlled facilitators to achieve better scalability and sustainability of team-based training in interprofessional education.

**Trial Registration:**

ClinicalTrials.gov NCT04330924; https://clinicaltrials.gov/ct2/show/NCT04330924

## Introduction

The relationship between communication and patient safety has emphasized the importance of team training for health professional education [1]. Improved communication among health care teams has been associated with better quality in patient care and enhanced patient satisfaction [2]. In contrast, failures in communication have been found to be the cause of adverse patient events and negative health outcomes [3,4]. Effective communication remains a challenge between nurses and physicians due to training differences and embedded workplace cultures [5,6]. In an earlier study on collaborative practices between nurses and junior physicians, the physicians reported insufficient information conveyed by ward nurses about changes in patients’ conditions, whereas the ward nurses reported a lack of direct communication from physicians about changes in patient treatment plans. The study thereby called for interprofessional education around effective communication strategies that focus on open communication, shared information, decision making, and mutual respect [7].

There is a general consensus among health care professionals and educators that interprofessional team training needs to commence at the preregistration level and continue into workplace practice [1,3]. An evidence-based review has called for educators to incorporate tools such as Identity, Situation, Background, Assessment, and Recommendation (ISBAR) for information exchange between health care providers into interprofessional team training [8]. These tools create a shared mental model—team members’ shared understanding of relevant knowledge—for teams to communicate and enable the exchange of information between team members to facilitate decision making. The integration of these tools into interprofessional education has shown to improve students’ perceptions of interprofessional collaboration and confidence in communicating effectively with other team members [9]. However, these evaluations were based on self-reporting questionnaires and may not reflect actual team performances. In addition, such teaching has been primarily within uni-professional groups of medical and nursing students as there were challenges in bringing them together to learn structured communication interprofessionally [8].

Simulation, which is commonly used as a teaching method for health care team training, provides experiential opportunities in patient-care situations for teams to work together, practice their roles, and develop a shared understanding of a team and its associated tasks [9]. According to Weaver [4], team training programs are most effective with integrated learning activities that use tools to support teamwork practices. Previous studies have provided evidence on the effectiveness of incorporating simulations with communication tools for improving medical and nursing students’ communication skills and teamwork attitudes [10,11]. However, these studies were limited, and few evaluated performance outcomes from the preregistration team training. In addition, logistical issues in organizing simulation training, including simulation facilities and scheduling, have proved to be challenging particularly among preregistration health care students whom are often trained in different institutions [10]. Simulations in a virtual environment can overcome time and space constraints and are gaining popularity among health care educators. Being accessible anytime and anywhere, virtual simulations provide the flexibility to be integrated into curricula and have the scalability to train large numbers of learners.

With advances in computer learning technology, virtual reality provides a viable platform for team-based simulation training that can address logistical challenges implicit in traditional simulation, such as physical location availability and scheduling for different groups of students [12]. We developed a computer-based virtual reality known as CREATIVE (Create Real-time Experience and Teamwork in Virtual Environment), where users can create physical and social presences using avatars in a 3D virtual hospital environment. CREATIVE was developed based on gaps identified from a systematic review on the use of multiuser virtual reality in health care education. The review called for the incorporation of theoretical models to inform the virtual learning process and the employment of more rigorous research including randomized controlled trials (RCT) to evaluate the impact of multiuser virtual reality on clinical performances. We applied theories including experiential learning and social constructivism to support the development and implementation of simulation learning in CREATIVE [12]. A pilot study was performed that showed the usability and feasibility of CREATIVE in supporting social interactions and collaborative practices among diverse groups of health care students [13]. Another study was conducted on CREATIVE that supported the integration of cognitive tools and virtual simulations to develop shared mental models for the delivery of optimal clinical teamwork [14]. In this study, by incorporating communication tools into a team training program, we aimed to evaluate the effectiveness of virtual reality in comparison with live simulations on medical and nursing students’ communication skill performances and teamwork attitudes.

## Methods

### Study Designs and Participants

We conducted a prospective RCT with a pretest-posttest study design after our study was approved by the institutional review board. We used social media to recruit volunteers who were undertaking their third or fourth year of medicine or nursing courses in a local university. This group of students was targeted as they had undertaken acute care management in their respective courses. We calculated the sample size based on a medium effect size of 0.50 for an independent sample *t* test [15]. Cohen’s (1992) [15] sample size table reported that a sample size of 64 participants per group (total participants=128) would be sufficient to achieve a predefined power of 0.80 at a 5% level of significance (2-sided) in detecting a medium effect size. Divided by health care courses taken and year of study, the participants were randomly assigned to a virtual or simulation group. They were grouped into interprofessional teams of 2 medical students and 2 nursing students to undertake the study intervention. Each team was facilitated by a simulation-trained faculty member and a simulated patient.

### Study Interventions

The participants in the virtual and live simulation groups underwent 3 hours of team training on nurse-physician communication conducted in the university’s center for health care simulation. After obtaining written informed consent from participants, they were directed to a 20-minute computer-based lesson on communication skill strategies. We adapted the strategies from the Team Strategies and Tools to Enhance Performance and Patient Safety (TeamSTEPPS) curriculum, which included ISBAR; Concerned, Uncomfortable, and Safety (CUS); feedback to acknowledge; callout; and check back [16].

**Figure 1:**
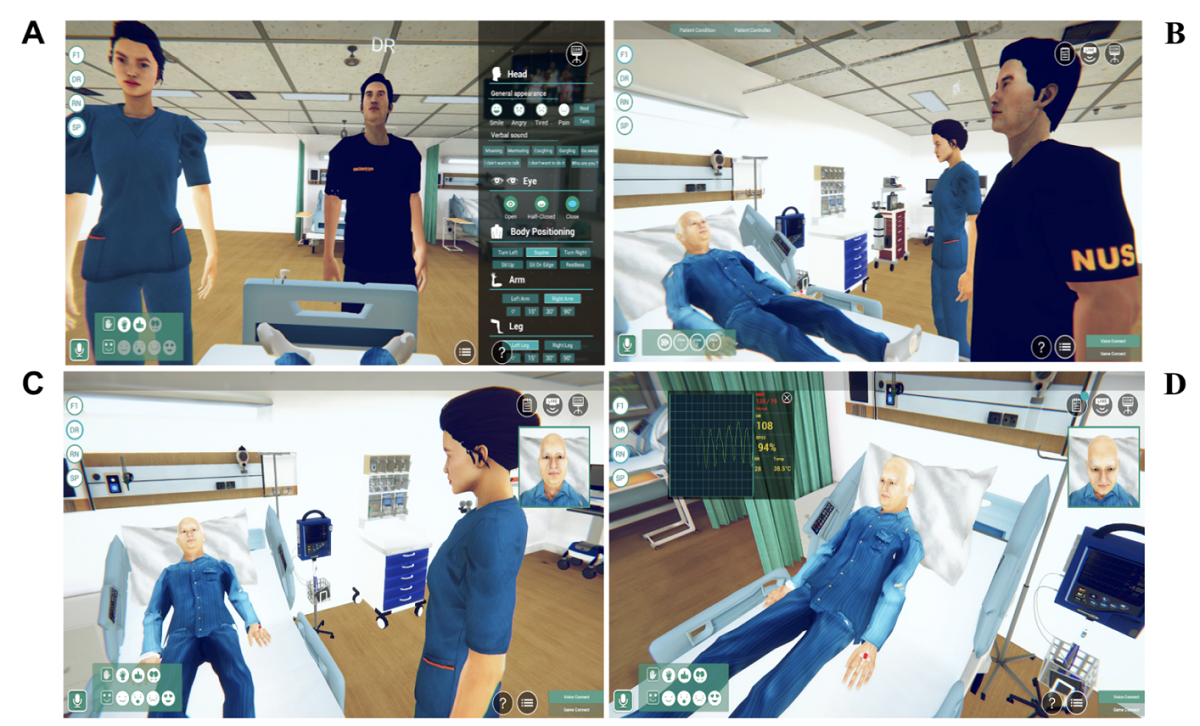
Viewpoints of different users. A: simulated patient’s view; B: facilitator’s view; C: medical student’s view; D: nursing student’s view.

The participants in the virtual group remained in the computer laboratory to undertake the virtual reality simulation. Together with their teammate, a facilitator, and a simulated patient, they logged into the 3D virtual environment using their avatar roles. Figure 1 illustrates their viewpoints when interacting with one another and the virtual environment. The virtual groups were given an orientation in which they learned to navigate between tutorial room and ward settings, talk among themselves using headsets, and perform assessments on the patient avatar. The participants in the live simulation group were brought into a simulated ward setting for their simulations and received an orientation on the ward setup, the equipment, and a standardized patient.

Table 1 describes the tasks performed by the participants in virtual reality and live simulations. After an orientation, the participants and facilitators gathered in a physical or virtual tutorial room for a briefing on the learning objectives and activities. In both groups, the participants in each team were randomly paired up (1 medicine student and 1 nursing student) and took turns role-playing and observing to participate in two simulation scenarios. The first scenario simulated a morning round situation of a postoperative patient with sepsis conditions, while the second scenario involved the same patient whose condition had deteriorated into septic shock. The participants were given time to read the case history before commencing each scenario. Each scenario began with the nursing participant performing a nursing assessment on the patient, followed by communicating to the medical participant. In these scenarios, the nursing participants were expected to use communication strategies (eg, ISBAR, CUS, check back) to communicate the assessment findings to the medical participants. The medical participants were expected to also perform physical assessments and apply communication strategies by acknowledging the nurse’s concerns and communicating treatment plans using the callout strategy. The clinical findings and responses displayed by both the patient avatar and the standardized patient were similar based on the prepared scripts. Each scenario lasted about 15 to 20 minutes and was followed up by a 30-minute debriefing. The debriefing was led by a facilitator using a facilitator guide that focused on the application of communication strategies.

**Table 1:** Comparison of tasks performed by the participants in virtual reality and live simulations.

Tasks	Virtual Reality	Live Simulation
Orientation	The participants navigated in the virtual environment, tested their headsets for communication, and became orientated with clickable icons and objects.	The participants were orientated to the physical ward setting, the equipment, and a simulated patient.
Briefing	The participants were briefed in real time using headsets by a facilitator avatar in the virtual tutorial room.	The participants were briefed face-to-face by a live facilitator in the physical room setting.
Reading case history	The participants read the case scenario by clicking the laptop in the virtual tutorial room and the COW^a^ in the virtual ward.	The participants read the case scenario from a case file placed in the tutorial room and ward setting.
Patient assessment	The participants clicked on the body parts of the patient avatar and on monitoring machines.	The participants performed physical assessments on the live simulated patient and used monitoring machines.
Interventions and treatments	The participants clicked on the appropriate trolley or equipment.	The participants performed hands-on interventions or treatments.
Communication	The participants communicated with each other using headsets and clickable gestures.	The participants communicated with each other face-to-face.
Debriefing	The participants undertook a group debriefing in the virtual tutorial room with the facilitator avatar.	The participants undertook a face-to-face group debriefing in the physical room with the live facilitator.

^a^COW: computer on wheels.

### Data Collection and Instrument

We administered the Attitudes Toward Interprofessional Health Care Team (ATHCT) and Interprofessional Socialization and Valuing Scale (ISVS) questionnaires before (baseline), immediately after (posttest), and 2 months after (follow-up) the simulation training to measure teamwork attitudes. The ATHCT, a 14-item tool using a 5-point scale validated by Kim and Ko [17], was adopted to measure the participants’ attitudes toward working in interprofessional health care teams. We obtained a high internal consistency in this study, with a Cronbach alpha of .83. The ISVS, a 24-item questionnaire using a 7-point scale developed by King et al [18], was used to capture the participants’ beliefs, behaviors, and attitudes in interprofessional socialization. This study reported a high Cronbach alpha of .86.

After the study intervention, the participants from both groups were brought to a room for team-based simulation assessments. They were assigned to work in pairs (medical and nursing) based on their earlier simulation teams. The teams were given a case history to read and an orientation of the simulation room with a manikin setup. The test scenario began with the nursing student performing the nursing assessment and management of the manikin, which was displaying signs and symptoms of deterioration, and calling the doctor. Upon the medical participant’s arrival, they worked as a team to manage the deteriorating patient. The team-based simulation lasted about 15 minutes and the entire process was recorded. We sent the recorded videos for rating to a clinician and an academic staff member whom were blinded to the groupings. The assessors rated the team communication performances independently using a validated team communication scale. We developed the scale using a 7-item checklist with a 5-point scale and a global rating item based on observable team communication skills between the nurse and doctor. We sent the scale for content validation to an interprofessional team of 4 medicine and nursing academics and clinicians. We computed the interrater reliability across 2 raters who scored the video-recorded performances independently using the validated scale. A high intraclass correlation coefficient (ICC) of 0.96 (95% CI 0.93-0.97) was reported, indicating good interrater agreement.

### Data Analysis

We applied descriptive statistics, chi-square tests, and *t* tests to analyze the demographic characteristics of the study population. We computed a paired sample *t* test to examine significant changes between the baseline and posttest performance scores and an independent sample *t* test to determine differences in the posttest scores between the groups. We performed a repeated measures analysis of variance (ANOVA) for the ATHCT and ISVS scores.

## Results

### Demographic Characteristics

Although we targeted 128 participants, only 120 participants completed the study. The majority were female (81, 67.5%), Chinese (105, 87.5%), and an average of 22.17 years of age (SD 2.07). There were no significant differences in the baseline characteristics, including age (*P*=.06), gender (*P*=.17), year of study (*P*=.85), and ethnicity (*P*=.94) between the virtual and simulation groups (Table 2). This supported the homogeneity of the participants between the 2 groups.

**Table 2:** Demographic characteristics.

Characteristics		Overall (N=120)	Virtual (N=60)	Simulation (N=60)		*P* value
Age (years), mean (SD)		22.17 (2.07)	21.82 (1.07)	22.53 (2.70)		.06
**Gender, n (%)**					.17	
	Male	39 (32.5)	16 (26.7)	23 (38.3)		
	Female	81 (67.5)	44 (73.3)	37 (61.7)		
**Course, n (%)**					>.99	
	Medicine	60 (50.0)	30 (50.0)	30 (50.0)		
	Nursing	60 (50.0)	30 (50.0)	30 (50.0)		
**Year of study, n (%)**					.85	
	Third year	41 (34.2)	21 (35.0)	20 (33.3)		
	Fourth year	79 (65.8)	39 (65.0)	40 (66.7)		
**Ethnicity, n (%)**					.94	
	Chinese	105 (87.5)	53 (88.3)	52 (86.7)		
	Indian	9 (7.5)	4 (6.7)	5 (8.3)		
	Malay	6 (5.0)	3 (5.0)	3 (5.0)		

### Team Communication Performance

The team-based simulation assessment revealed no significant differences in the overall communication performance posttest scores (*F*_2,58_=1.46, *P*=.29, eta^2^=0.33) between the virtual (mean 22.60, SD 5.31) and simulation groups (mean 23.97, SD 4.55). There were also no significant differences in the total checklist posttest scores (*F*_2,58_=3.654, *P*=.29, eta^2^=0.28) and global posttest scores (*F*_2,58_=1.56, *P*=.29, eta^2^=0.33) between the groups.

### Teamwork Attitudes

At the baseline, there were no significant differences in the ATHCT (*P*=.33) and ISVS (*P*=.45) scores between the virtual and simulation groups, supporting the homogeneity of the participants between the groups. After the team training, there was a significant increase in the ATHCT and ISVS posttest scores from the baselines scores for both groups. There was also a significant increase in the follow-up ISVS scores from the baseline scores for the virtual group (*P*=.047) but not for the simulation group (*P*=.14). No significant differences between the baseline and follow-up ATHCT scores were found for both groups (Table 3).

Using repeated-measure ANOVA, there were no significant differences in the trend between the virtual and simulation groups for both the ATHCT (*P*-interaction=.58, eta^2^=0.005) and ISVS (*P*-interaction=.61, eta^2^=0.004) scores. There were also no significant differences in the ATHCT (F_2,118_=0.507, *P*=.48, eta^2^=0.004) and ISVS (F_2,118_=0.335, *P*=.56, eta^2^=0.003) scores over the 3 time points between the virtual and simulation groups.

**Table 3:** Comparison of teamwork attitude scores on Interprofessional Socialization and Valuing Scale and Attitudes Toward Interprofessional Health Care Team.

Group		Baseline	Posttest	*P* value	Baseline	Follow-up test	*P* value
**ISVS^a^ (teamwork attitude score)**							
	Virtual, mean (SD)	131.78 (15.81)	142.92 (14.88)	<.001	131.78 (15.81)	136.62 (6.43)	.047
	Simulation, mean (SD)	134.03 (16.31)	143.95 (15.47)	<.001	134.03 (16.31)	137.45 (15.90)	.14
	*T* test (2-tailed)	0.45	0.71	N/A^b^	0.45	0.78	N/A
**ATHCT^c^ (teamwork attitude score)**							
	Virtual, mean (SD)	58.22 (5.78)	60.05 (5.41)	.004	58.22 (5.78)	58.27 (6.57)	.95
	Simulation, mean (SD)	57.12 (6.58)	59.95 (6.65)	<.001	57.12 (6.58)	57.17 (7.26)	.96
	*T* test (2-tailed)	0.33	0.93	N/A	0.33	0.39	N/A

^a^ISVS: Interprofessional Socialization and Valuing Scale.

^b^N/A: not applicable.

^c^ATHCT: Attitudes Toward Interprofessional Health Care Team.

## Discussion

### Principal Findings

The outcomes in this RCT study did not show the inferiority of computer-based virtual reality on teamwork attitudes and communication skill performances when compared with live simulations. These outcomes were consistent with earlier studies [19,20], providing more evidence to support its potential use in substituting conventional team-based simulations. In this virtual reality team training, we incorporated design elements that mimicked live simulations for the experiential learning and the ability to support multiusers, including students, a simulated patient, and a facilitator to come together for collaborative learning.

Despite the different learning mediums, both offered similar learning strategies, including experiential and collaborative learning, that allowed medical and nursing students to practice the use of communication tools through roleplay exercises and engage in reflective practices through interactions with one another and their facilitators [12]. These learning activities are underpinned by the theories of experiential learning and social constructivism. Kolb’s experiential learning theory supports the learning mechanism of roleplaying and debriefing, and the theory of social constructivism emphasizes social interactions underlying collaborative learning. Thus, we call upon educators and instructional designers to apply these theories to support the implementation of simulation learning in virtual reality learning environments [12].

We believe that teaching teamwork at the preregistration level is important but has limited impact on organization and patient outcomes. Our long-term evaluation of virtual reality on the retention of learning was limited to measuring changes in interprofessional attitudes 2 months after the intervention, with significant improvements in attitudes toward interprofessional socialization but not in attitudes toward interprofessional health care teams. Caution has to be taken on the validity of self-reported measures. Nonetheless, the opportunities to engage in social interactions in virtual reality could have improved students’ attitudes toward interprofessional socialization. Social interactions between the medical and nursing students were also facilitated using structured communication tools, which could have promoted open communication, shared information, and decision-making [11]. One advantage of virtual reality over live simulations was its ability to allow anonymous social interactions in its environment, which may cause less social anxiety and stress for students [13,21]. We believe that positive attitudes toward interprofessional socialization are important in building future collegial working relationships between nurses and physicians.

The lack of improvement in attitudes toward interprofessional health care teams after the study interventions for both virtual and simulation groups could be due to the influence of workplace realities faced by students during their clinical practicums, wherein issues of powers, structures, and systems may limit health care teams from collaborating effectively. This highlights the importance of addressing workplace systems and structures, including the implementation of workplace team training [22]. Future studies should investigate how virtual reality can contribute to postqualification workplace-based team training. This may include the use of virtual reality and workplace simulations as a blended learning approach to optimize interprofessional learning [23].

Although we did not compare the cost and resources of the virtual and simulation interventions, we recognized the challenges for sustainability and scalability. The existing virtual reality serves as a flexible and practical platform to bring everyone together, but its scalability is constrained by human-controlled avatars. The recruitment and training of people for the avatar role of a patient were found to be less convenient and costly. In addition, as a result of unequal cohort size across different health care courses (ie, nursing students vs medical students), it is unlikely that all health care students will form interprofessional teams. We plan to develop and integrate artificial intelligence (AI) in virtual reality to replace the roles of a simulated patient and even a medical doctor. With evolving AI technology, the goal is to design AI agents that can interact with users in a natural, humanlike way such as via natural language processing [24]. The AI can cohabit virtual worlds with people and facilitate deep engagement of learning that could potentially improve learning outcomes [25]. A recent systematic review on AI in medical education highlighted the potential use of AI in virtual reality and called for more evidence to justify its effectiveness [26]. Therefore, future studies can examine the effectiveness of AI-controlled agents compared to human-controlled avatars in virtual reality simulations.

This study used the topic of sepsis and septic shock for the application of TeamSTEPPS communication strategies as the curriculum’s content. However, the virtual platform was created with expandability in mind, allowing other medical conditions such as cardiac arrest and other teamwork curricula to be programmed and applied in the CREATIVE platform. We have collaborated with other local higher education institutions to develop geriatric-related cases using the CREATIVE platform to support teamwork in interprofessional rounding among diverse students of health care professions[11]. We hope to open up the possibility of international collaborative learning using this platform and to study its effectiveness.

### Limitations

Although we tested the students’ team performances and attitudes using simulation-based assessments and validated tools, the quality of evidence was limited by an immediate posttest on team performance and self-reported attitude questionnaires. Future studies can provide more evidence on team performance by examining the baselines and retentions of team performances. The study intervention was carried out at a single location, the university’s simulation center, to capture the participants’ team performances using simulation-based assessments. Future implementation can occur across different physical locations, including access from home. Future studies can be conducted to gain insights into the learning process of users and identify any potential usability issues across distant locations.

### Conclusion

Interprofessional team training using simulations has proved to be logistically challenging to implement at the preregistration level due to difficulties in bringing together different groups of health care students. We implemented doctor-nurse communication team training by incorporating communication strategies from the TeamSTEPPS curriculum into computer-based virtual reality for undergraduate medical and nursing students. Our study outcomes did not show any difference between virtual and live simulations in terms of teamwork attitudes and communication skill performances, which supports the potential use of virtual reality to substitute conventional team-based simulation training. Further developments and evaluations can use AI technology to replace costly human-controlled avatars to achieve better scalability and sustainability of team-based training in interprofessional education. With its scalability and practicality, virtual reality serves as a promising learning strategy to prepare students to be part of a future collaborative workforce that can provide safe and quality patient care.

## Appendix

Multimedia Appendix 1 CONSORT-EHEALTH checklist (V 1.6.1).

## Abbreviations

AI: artificial intelligence
ANOVA: analysis of variance
ATHCT: Attitudes Toward Interprofessional Health Care Team
CREATIVE: Create Real-Time Experience and Teamwork in Virtual Environment
CUS: Concerned, Uncomfortable, and Safety
ISBAR: Identity, Situation, Background, Assessment, and Recommendation
ISVS: Interprofessional Socialization and Valuing Scale
RCT: randomized controlled trial
TeamSTEPPS: Team Strategies and Tools to Enhance Performance and Patient Safety.
